# Supplementary material for: The interactome of intact mitochondria by cross-linking mass spectrometry provides evidence for coexisting respiratory supercomplexes
Source: Mol Cell Proteomics. 2017 Dec 8;17(2):216–32. doi: 10.1074/mcp.RA117.000470 (PMC5795388; doi:10.1074/mcp.RA117.000470)
Supplement: Supplemental Data [file supp_RA117.000470_133922_0_supp_23978_fzffwf.zip › spectra_annotation/mito_spectra_annotation/419-1-3-1-10-1.pdf]

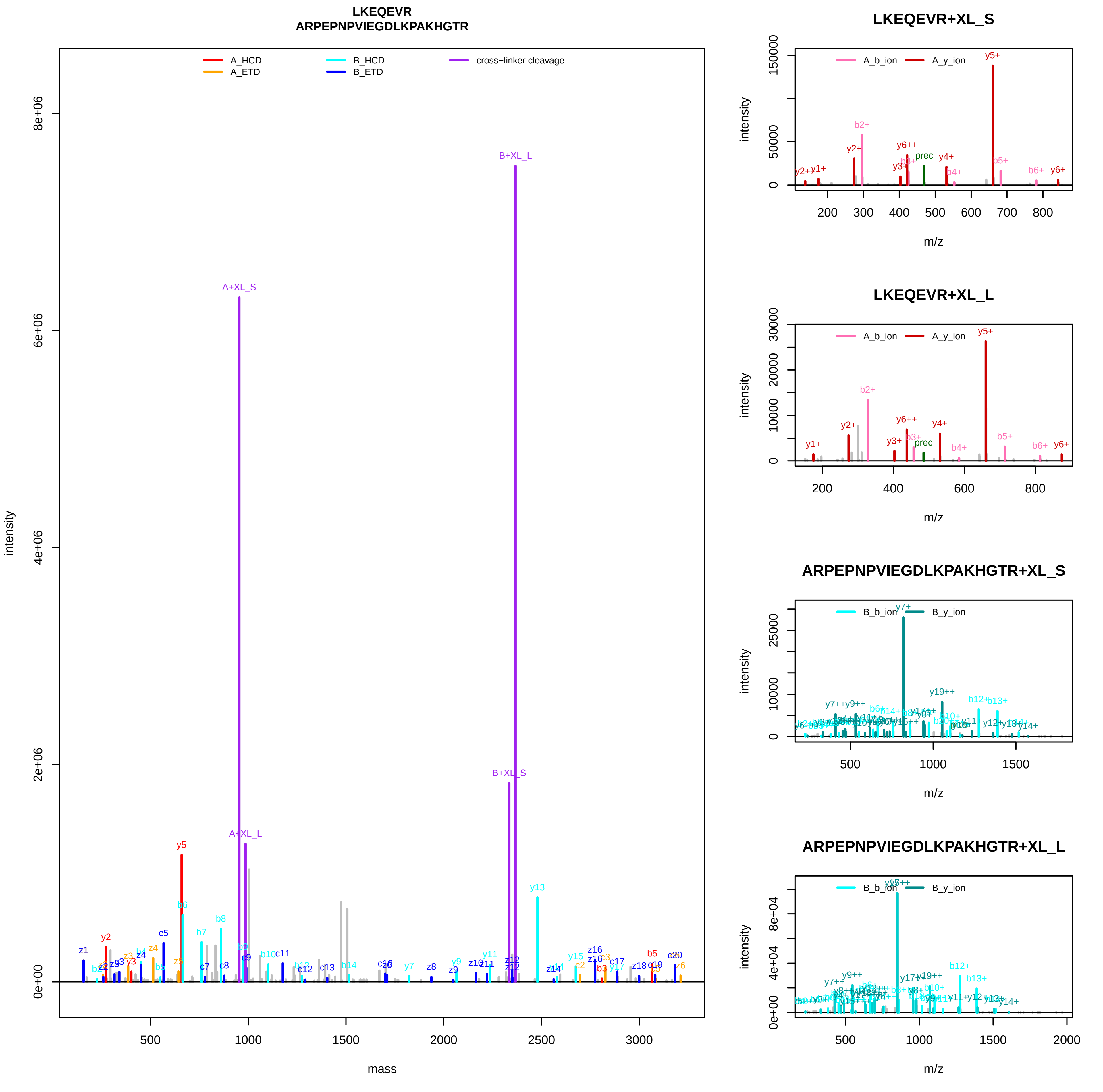

LKEQEVR
ARPEPNPVIEGDLKPAKHGTR
A_HCD
A_ETD
B_HCD
B_ETD
cross−linker cleavage
B+XL_L
A+XL_S
B+XL_S
A+XL_L
y5
y13
b6
b8
b7
c5
y2
z1
c11
b10
b14
y11
y9
y15
z16
c17
c20
b5
c19
z6
z14
z12
z11
z10
z9
z8
y7
c16
c13
c12
c9
b9
c8
c7
z4
b4
z3
z2
y3
z5
b2
c2
b3
z18
y17
0e+00
2e+06
4e+06
6e+06
8e+06
intensity
500
1000
1500
2000
2500
3000
mass
LKEQEVR+XL_S
A_b_ion
A_y_ion
y5+
b2+
y2+
y6++
b3+
prec
y4+
y3+
b5+
b4+
b6+
y6+
y2++
y1+
0
50000
150000
intensity
200
300
400
500
600
700
800
m/z
LKEQEVR+XL_L
A_b_ion
A_y_ion
y5+
b2+
y2+
y6++
y4+
y3+
b3+
prec
b5+
b4+
b6+
y6+
y1+
0
10000
20000
30000
intensity
200
400
600
800
m/z
ARPEPNPVIEGDLKPAKHGTR+XL_S
B_b_ion
B_y_ion
y7+
y19++
b12+
b13+
y7++
y9++
y12+
y13+
y14+
y11+
0
10000
25000
intensity
500
1000
1500
m/z
ARPEPNPVIEGDLKPAKHGTR+XL_L
B_b_ion
B_y_ion
y15++
y17++
y19++
b12+
b13+
y7++
y9++
y8+
y11+
y12+
y13+
y14+
0e+00
4e+04
8e+04
intensity
500
1000
1500
2000
m/z
